# Dietary components as epigenetic-regulating agents against cancer

**DOI:** 10.7603/s40681-016-0002-8

**Published:** 2016-02-23

**Authors:** Ling-Chu Chang, Yung-Luen Yu

**Affiliations:** 1 Chinese Medicinal Research and Development Center, China Medical University Hospital, 404 Taichung, Taiwan; 2 Graduate Institute of Cancer Biology, China Medical University, 404 Taichung, Taiwan; 3 Center for Molecular Medicine, China Medical University Hospital, 404 Taichung, Taiwan; 4 Department of Biotechnology, Asia University, 413 Taichung, Taiwan

**Keywords:** Dietary component, Epigenetic modification, Cancer

## Abstract

Carcinogenesis is a complicated process that involves the deregulation of epigenetics resulting in cellular transformational events, such proliferation, differentiation, and metastasis. Epigenetic machinery changes the accessibility of chromatin to transcriptional regulation through DNA modification. The collaboration of epigenetics and gene transcriptional regulation creates a suitable microenvironment for cancer development, which is proved by the alternation in cell proliferation, differentiation, division, metabolism, DNA repair and movement. Therefore, the reverse of epigenetic dysfunction may provide a possible strategy and new therapeutic targets for cancer treatment. Many dietary components such as sulforaphane and epigallocatechin- 3-gallate have been demonstrated to exert chemopreventive influences, such as reducing tumor growth and enhancing cancer cell death. Anticancer mechanistic studies also indicated that dietary components could display the ability to reverse epigenetic deregulation in assorted tumors via reverting the adverse epigenetic regulation, including alternation of DNA methylation and histone modification, and modulation of microRNA expression. Therefore, dietary components as therapeutic agents on epigenetics becomes an attractive approach for cancer prevention and intervention at the moment. In this review, we summarize the recent discoveries and underlying mechanisms of the most common dietary components for cancer prevention via epigenetic regulation.

## 1. Introduction

Epigenetic alternation is referred to the change in gene expression without changing in DNA sequence. Epigenetic modifications include DNA methylation, histone modification (acetylation, methylation, and phosphorylation), and microRNA expression, playing a critical role in many cellular processes [1-4]. The initiation and progression of cancer have been proved as the results of the accumulation of genetic mutations which lead to aberrant cellular functions [3]. Genetic mutations may cause the activation of oncogenes and the inactivation of tumor suppressor genes. In recent decades, dietary components which are widely present in daily dietary, have been proved to exhibit a beneficial effect in cancer prevention and treatment. They reverse epigenetic deregulation by modulation of DNA methylation and histone modification, as well as alternation of microRNA expression [4-7]. This review will introduce some common dietary components possessing anticancer effects and elucidate their mechanisms.

## 2. Epigenetic modifications

### 2.1. DNA methylation

In mammalian cells, DNA methylation almost occurs at the 5’ position of cytosine residues within cytosine-phosphate-guanine (CpG) island dinucleotides [8], which is catalyzed by DNA methyltransferases (DNMTs) and *S*-adenosyl-methionine (SAM) as the methyl donor [9]. So far, five DNMTs have been identified, including DNMT1, DNMT2, DNMT3a, DNMT3b, and DNMT3L, and only three possess catalytic methyltransferase activity (DNMT1, DNMT3a, and DNMT3b) [9, 10]. DNMT1 is responsible for maintaining existed methylation patterns during DNA replication by adding methyl groups to corresponding daughter strands at the hemimethylated CpG sites. DNMT3a and DNMT3b carry out *de novo* methylation and preferentially target unmethylated CpG sites. DNMT3L lacks intrinsic methyltransferase activity, but can facilitate the methylation of retrotransposons by interaction with DNMT3a and 3b [9]. DNMT3L also recognizes the unmethylated lysine 4 of histone H3 to enhance the *de novo* methylation of DNA by the recruitment and activation of DNMT3a [11]. In normal cells, CpG islands of most genes are maintained in unmethylated, allowing RNA polymerase II to bind and transcription to proceed. Cancer cells often maintain in global hypomethylation status and hypermethylation of promoter CpG islands. Global hypomethylation can lead to chromosomal instability and mutations and result in the re-activation of oncogenes. DNA hypermethylation of the promoter CpG islands of tumor suppressor genes causes their transcriptional silencing [12]. Hence, aberrant promoter methylation allows the cancer cells to have a potent ability in growth and invasion [2, 3, 9].

### 2.2. Histone modifications

DNA is tightly compacted by histone proteins [13]. Octamer of histone proteins (two molecules of each H2A, H2B, H3, and H4) are wrapped with 146 bp of DNA to form a nucleosome. Histone proteins regulate the chromatin dynamic *via* changing chromatin structure by electrostatic charge alternation or providing protein recognition sites for modification [14, 15]. Histone modification occurs at the N-terminal tail, which subsequently affects DNA processes, including transcription, DNA repair, and DNA replication. Histones can be modified post-translationally by acetylation, methylation, phosphorylation, sumoylation, biotinylation, ubiquitination, ADP-ribosylation, deamination, proline isomerization, and propionylation [16-19].

Chromatin can exist in two different states, closed configuration (heterochromatin) and open configuration (euchromatin). The closed chromatin configuration is hard to access for the transcriptional machinery and generally harbor transcriptionally inactive genes. Histones (mainly H3) can be acetylated at lysine residues of N-terminal tails, which regulates chromatin into open or closed form. Acetylation neutralizes the positive charge of histones followed by dis-association from the negatively charged DNA backbone, leading to the open chromatin structure and making accessible to transcriptional machinery. Thereby, histone acetylation is generally associated with transcriptional activation [17-19]. Histone acetylation is carried out by histone acetyltransferases (HATs) and histone deacetylases (HDACs). HATs catalyze histone acetylation, while HDACs catalyze deacetylation by the removal of acetyl group, resulting in compact chromatin configuration and restricting the transcription factor access, and thereby inhibiting gene expression [18, 19]. HATs are classified into five families, Gcn5-related *N*-acetyltransferase (GNAT), MYST, p300/CBP, nuclear receptor coactivators (SRC), and TAFII250 families [20]. So far, eighteen human HDACs have been identified and divided into four classes, including Class I, II, III, and IV [20].

Methylation is also involved in histone modification. Unlike acetylation, methylation does not change the protein charge [16, 21]. Histone methylation occurs in lysine and arginine residues, which are catalyzed by histone lysine methyltransferase and histone arginine methyltransferase respectively [22]. Until now, twenty-four sites of methylation of histone are recognized, including 17 lysine residues and 7 arginine residues [23]. Lysine residues can be mono-, di-, or trimethylated, whereas arginine residues are mono- or dimethylated. The methylation of lysine is responsible for the alternation of chromatin structure [21]. Monomethylation of histone 3 (H3) at lysine 4 (H3K4me1) and trimethylation of H3 at lysine 4 (H3K4me3) are associated with transcriptional activation, whereas trimethylation of histone H3 at lysine 9 (H3K9me3), lysine 20 (H3K20me3), and lysine 27 (H3K27me3) are correlated with transcriptional inactivation [14]. Dysregulation in polycomb repressive complex 2 -mediated histone H3K27me3 is frequently observed in many types of cancers [14]. Enhancer of zeste homolog 2 (EZH2) is the catalytic component of PRC, which selectively trimethylates H3K27. Evidences suggest that overexpression of EZH2 is highly associated with cancer progression and outcome in different cancers. Therefore, EZH2 is regarded as a therapeutic target for cancer treatment [24]. Histone demethylation is catalyzed by histone demethylases, including LSD and JMJC families [25].

Histone phosphorylation is regulated by kinases and phosphatases *via* adding or removing phosphate groups from the hydroxyl group of serines, threonines, and tyrosines of histone N-terminal tails. Phosphorylation of histone also alters the charge of histone, resulting in the structure alternation of chromatin [26].

### 2.3. Non-coding microRNAs

MicroRNAs are small, noncoding regulatory RNAs ranging in size from 17 to 25 nucleotides, which are matured by Dicer/Drosha RNase form hairpin-structured precursors [27]. MicroRNAs post-transcriptionally inhibit gene expression by recognizing complementary target sites in the 3’-untranslatied regions of target mRNAs [28]. MicroRNAs play critical roles in cell proliferation and differentiation, cell cycle control, and cell death, as well as tumor development and metastasis [28, 29]. Aberrant microRNAs expression is observed in diverse cancers, and the expression varies in cancer phenotypes and stages. MiRNAs have been demonstrated their importance in *de novo* methylation of imprinted loci and act on mRNA of DNMTs and HDACs [30]. Each miRNA is capable of regulating the expression of many genes, allowing them to simultaneously regulate multiple cellular signaling pathways [28, 29]. Hence, miRNAs have the potential to be used as biomarkers for cancer diagnosis and prognosis, as well as therapeutic targets [31, 32].

## 3. Dietary components in cancer prevention and treatment

**Figure 1:**
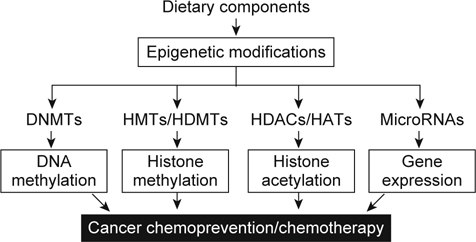

Many bioactive components in diets have been demonstrated to be effective in cancer prevention and intervention through epigenetic alternation [4-8]. In this review, we introduce some common dietary components and their epigenetic targets in cancer. Epigenetic modifications affected by dietary components are presented in Figure 1. Some bioactive components in diet and their epigenetic targets are summarized in Table 1.

### 3.1. Epigallocatechin-3-gallate (EGCG)

A huge number of studies implicated the anticancer properties of EGCG, a catechin isolated from green tea, with a positive correlation between green tea consumption and the inhibition on cancer [33, 34]. EGCG have a potent inhibitory effect on DNMT activities and DNA methylation in human cancer cells, including esophageal, colon, prostate, and breast cancer cells [35-37]. Treatment of EGCG leads to the demethylation of reactivation of *p16, Retinoic acid receptor (RAR)β, MGMT*, and *human mutL homolog 1 (hMLH1)*, and *glutathione S-transferase p (GSTP)* in human oesophageal cancer KYSE 510 cells [35]. The catechol group of catechins plays an important role in which these compounds exert their anticancer functions by inhibiting the methyltransferase activities. Catechol group is an excellent substrate for the methylation mediated by catechol-*O*-methyltransferase (COMT). COMTmediated methylation leads to the depletion of the methyl donor SAM and promotes the formation of *S*-adenosyl-_L_-homocysteine (SAH), inhibiting DNA methylation [38]. Thereby, these catechol- contained compounds inhibit methyltransferase activity by acceleration of COMT-mediated methylation, increasing the levels of SAH. Additionally, the molecular modeling studies indicated that EGCG can directly inhibit the DNMTs through the inhibitory interaction between the gallic acid moiety of EGCG and the catalytic sites of the DNMTs [36]. Furthermore, EGCG promotes the degradation of DNMT3a and HDAC3 [39], as well as the inhibition of HDAC activity [40]. In skin cancer cells, SCC-13 and A431, EGCG reduces the level of polycomb proteins including EZH2, EED, SUZ12, Mel18, and Bmil, which results in the reduction of H3K27me3 and H2AK119ub formation, as well as survival of cancer cells [41]. EGCG also have an effect on miRNAs in human cancer cells. In hepatocellular carcinoma HepG2 cells treated with EGCG, thirteen miRNAs are upregulated and forty-eight miRNAs are down-regulated [42].

**Table 1:** 

Natural products	Epigenetic modifications	Anticancer mechanisms	References
EGCG (green tea)	DNA methylation Histone methylation MicroRNAs	↑ *p16, RARβ, hMLH2, GSTP* ↓ DNMT3a, HDAC3, HDAC activity, EZH2, EED, SUZ12, Mel18, Bmi1, H3K27me3, H2AK119ub, Bcl-2, *miR-16*	35-42
Curcumin (turmeric)	DNA methylation Histone acetylation Histone methylation MicroRNAs	↑ HDAC1, HDAC4, HDAC5, HDAC8, SOCS1, SOCS3, *miR-15a, miR-16, miR-22, miR-26, miR-101, miR-146, miR-186, miR-200, miR-203, miR-192-5p/215, let-7* ↓ DNMT1 activity, HAT activity, DNMT1, DNMT3a, HDAC3, HDAC8, EZH2, miR-21, miR-34a, miR-199*	43-50
Resveratrol (grapes, berries, plum, peanuts)	DNA methylation Histone acetylation Histone methylation MicroRNAs	↑ DNMTs, MBD2, FOXC2, *PDCD1, PTEN, Dicer, mir-137, miR-663, miR-773* ↓ SIRT1, SIRT2, SIRT3, p300, eEF1A2, EZH2, *TGFβ, miR-17, miR-21, miR-25, miR-29a, mir-196a, miR-520h*	54-63
Quercetin (soybeans)	Histone acetylation MicroRNAs	↑ HAT activity, FasL ↓ HDAC activity, Sp, Survivin, *miR-21, miR-27*	64-69
Genistein (soybeans)	Histone acetylation Histone methylation MicroRNAs	↑ *PTEN, CYLD, p53, FOXO3a, miR-34a*, Notch-1 pathway ↓ SIRT activity, *miR-21*	70-75
Isothiocyanates (broccoli, cabbage, brussels sprouts, watercress, kale, cauliflower)	Histone acetylation Histone methylation MicroRNAs	↑ p300, p16, p21, Bax, *miR-17* ↓ HDACs, PCAF, EZH2, Bmi1, H3K27me3, cyclin B1, cyclin A, cyclin dependent kinase 1/2, *miR-20a, miR-27a, miR-17-5p*	76-89

### 3.2. Curcumin

Curcumin (diferuloylmethane), a yellow polyphenol from the rhizomes of *Curcuma longa*, is commonly used as spice and food coloring agent. The major components in the isolated curcuminoid complex are curcumin (approximately 80%), demethoxycurcumin (approximately 17%), and bisdemethoxycurcumin (approximately 3%) [43]. *Curcuma longa* has been used in traditional medicine in Asia for thousands of years. Recently, curcumin has been identified to exhibit antitumor and apoptosis-induction activity in a variety of human cancer cell lines [44-50]. Moreover, curcumin have also been applied in cancer treatment, including pancreatic cancer, multiple myeloma, and colorectal cancer [51]. Curcumin inhibits DNMT1 activity by covalently blocking the catalytic thiol group of Cys1226 binding site [52]. Prostate LNCaP cells treated with curcumin causes the demethylation of the CpG islands of the *NEUROG1* and *NRF1* genes. Curcumin also has the effects on the protein expression of HDACs, increasing the expression of HDAC1, 4, 5, and 8 and decreasing HDAC3 [44]. In leukemia K562 and HEL cells, curcumin elevates the expression of SOCS1 and SOCS3 *via* inhibiting HDAC8 expression to increase the acetylation of histone in the regions of *SOCS1* and *SOCS3* promoters [45]. Acetylation of the histone protein p300/CBPB and the non-histone protein p53 can be inhibited by curcumin through inhibition on HAT activity [53]. Furthermore, curcumin can enhance the anticancer effect on HDAC inhibitor, trichostatin A, in breast cancer cells SKBR3 and 435eB [46]. In MDA-MB-435 breast cancer cells, curcumin induces the downregulation of EZH2 expression through MAPK pathway [47]. In MCF-7 breast cancer cells and leukemia cells, curcumin upregulates the expression of *miR-15* and *miR-16*, resulting in Bcl-2 downregulation and apoptosis induction [49, 50]. In human pancreatic cells, curcumin increases *miR-22* and inhibits miR-199a*. In non-small cell lung cancer cells, the proapoptotic effects of curcumin depend on *miR-192-5p/215* induction [48]. MiRNAs and their targeted genes modulated by curcumin also comprise *miR-15a, miR-16, miR-21, mir-22, miR-26, miR-34, miR-101, miR-146, miR-200, miR-203,* and *let-7* [43].

### 3.3. Resveratrol

Resveratrol (3,5,4’-trihydroxystilbene) is a natural phytoalexin existing in several plants, such as grapes, berries, plums, and peanuts. Its anticancer effects are achieved by inhibiting the growth of cancer cells and inducing apoptosis [54, 55]. Resveratrol is capable of inhibiting the epigenetic silencing of the BRCA1 tumor suppressor protein [56]. Furthermore, resveratrol partially restores H3K9 mono-methylation, DNMTs, and MBD2 at the *BRCA1* promoter in MCF-7 cells [56]. Resveratrol shows to have the inhibitory effects on the class III HDACs, such as SIRT1, SIRT2, SIRT3, and p300 [57]. Resveratrol provides an effect in chemoprevention *via* SIRT1-encoded proteins in an *in vivo* skin tumor model [58]. In prostate cancer cells, resveratrol enhances p53 acetylation and apoptosis by inhibition of the metastasisassociated protein 1 (MTA)–nucleosome remodeling deacetylation (NuRD) complex [59]. In SW480 colon cancer cells, resveratrol affects the expression of *Dicer, PDCD1*, and *PTEN* by downregulation of oncogenic miRNAs, including *miR-17, miR-21, miR-25, miR-92a*, and *miR-196a*, while inhibiting the *TGFβ* by upregulation of *miR-663* [60]. In MCF-7 breast cancer cells, resveratrol upregulates *miR-663* and *miR-773*, which inhibits cell proliferation by inhibiting the eukaryotic translation elongation factor 1A2 (eEF1A2) at the mRNA and protein levels [61]. In CLI-5 and A549 lung adenocarcinoma cells, resveratrol downregulates *miR-520h* and induces *miR-520h*-mediated signaling pathway, resulting in the inhibition of forkhead box C2 (FOXC2) and the subsequent suppression of tumor metastasis in both *in vitro* and *in vivo* model [62]. In resveratrol-induced apoptosis and tumor suppression of neuroblastoma, EZH2 and H3K27me3 repression are mediated by *miR-137* [63].

### 3.4. Flavonoids

Flavonoids (bioflavonoids) are widely in plants, including fruits, vegetables, and beverages (coffee, tea, beer, wine, and juice) [64]. Flavonoids, such as quercetin, fisetin, and myricetin, have been shown to inhibit DNMT activity in different cancer cells [65]. Quercetin (3,5,7,3’,4’-pentahydroxyflavone) is the most abundant flavonoid in nature. In leukemia HL-60 cells, quercetin increases histone H3 acetylation which results in the promotion of the expression of FasL [66]. Quercetin exhibits the potential in the activation of HATs and the inhibition of HDACs, contributing to histone acetylation [66]. Furthermore, quercetin has an inhibitory effect on HAT activity and blocks TNF-α-induced acetylation and phosphorylation of histone H3 at the *IP-10* and *MIP-2* gene promoter in murine intestinal epithelial cells [67]. Quercetin also exerts an anticancer activity by regulation of miRNAs. The combination of quercetin and hyperoside significantly inhibits the invasion and migration of PC3 prostate cancer cells through downregulation of *miR-21* [68]. The combination of quercetin and hyperoside mediates *miR-27a* inhibition, inducing Sp-repressor ZBTB10 increase which inhibits Sp and survivin, leading to apoptosis of 786-O renal cancer cells [69].

Isoflavones are derived primarily from soybeans, including genistein, daidzein, and glycitein. Genistein inhibits the proliferation, invasion, and metastasis of cancer cells [70]. In addition, genistein has been showed to have a chemopreventive effect against various types of cancer cells, including prostate, esophageal, and colon cancer. Genistein is a phytoestrogen which binds to α and β estrogen receptors and regulates the intracellular signaling pathway to mimic the actions of endogenous estrogen, 17β-estradiol [71]. In esophageal and prostate cancer cells, genistein reverses aberrant DNA methylation which results in reactivation tumor repressor genes, including as *p16* ^INK4A^, *RARβ, MGMT, phosphatase and tensin homolog (PTEN), cylindromatosis (Turban tumor syndrome, CYLD)* by inhibiting DNMT activity [72]. In prostate cancer cells, genistein increases the acetylation of H3K9 at *p53* and *FOXO3a* promoter through inhibition of SIRT1 activity [73]. Genistein also inhibits *miR-21* in A-498 renal cancer cells growth and tumor growth in xenograft model through cell cycle arrest and apoptosis induction [74]. In pancreatic cancer cells, genistein inhibits cell growth and induces apoptosis through the up-regulation of *miR-34a* and Notch-1 signaling pathway [75].

### 3.5. Isothiocyanates

Isothiocyanates (ITCs), metabolites of glucosinolates, are found in cruciferous vegetables, such as broccoli, cabbage, brussels sprouts, watercress, kale, and cauliflower, which provide a remarkable anticancer effect on pancreatic, prostate, ovarian, and breast cancers [76, 77]. The known potent anticancer effects of ITCs are from allyl isothiocyanate (AITC), benzyl isothiocyanate (BITC), phenethyl isothiocyanate (PITC), and sulforaphane. The anticancer activities of ITCs can be divided into chemopreventive and chemotherapeutic effects. ITCs have a potential in inhibition of carcinogenesis *via* acting on detoxification, inflammation, apoptosis, and cell cycle, as well as epigenetic regulation [77]. ITCs are found to inhibit tumorigenesis through inhibition of HDACs [78, 79]. The inhibition of HDAC activity also contributes to the increase of tumor suppressor gene p21^WAF1^ and the pro-apoptotic gene Bax [80]. In myeloma cells, PITC can inhibit HDACs and induce DNA demethylation of tumor suppressor *p16* gene, which leads to its reactivation [81]. In HL-60 leukemia cells, PITC mediates cell growth arrest which is associated with the reduction of HDAC activity and increase of acetyltransferase p300 and p21^WAF1^ acetylation [82]. In hyperplastic (BPH1) and prostate cancer cells (LNCaP and PC3), sulforaphane induces cell cycle arrest and apoptosis by inhibition of HDAC activity and class I and II HDAC proteins, as well as increase of H3 acetylation followed by induction of *p21* ^WAF1^ expression [83]. The inhibition of HDAC is related to sulforaphane metabolite sulforaphanecysteine that can fit the enzyme pock and form a bidentate ligand through the interaction of α-carboxyl group of the cysteine moiety and buries zinc atom [79]. *In vivo* data showed that administration of sulforaphane to *Apc* ^min^ mice can inhibit intestinal tumors and increase histone acetylation of global DNA and the promoter regions of *p21* ^WAF1^ and *Bax* [84]. PITC has been proved to exert a lung chemopreventive effect by modulated the cigarette smokeinduced alternations on miRNA expression. Furthermore, the combination of PITC and indole 3-carbinol reverses the effects of cigarettes on these miRNAs [85, 86]. In prostate cancer cells, PITC inhibits androgen receptor by enhancing *miR-17* mediated suppression of p300/CBP-associated factor (PCAF), a co-regulator of androgen receptor [87]. Sulforaphane treatment causes the reduction of EZH2 and Bmi-1 expression in SCC-13 skin cancer cells and also reduces H3K27me3, which is associated with the accumulation of cells at G_2_/M phase, the decrease of cyclin B1, cyclin A, cyclin dependent kinases (CDK) 1/2, and the increase of p21^WAF1^ expression [88]. Additionally, PITC modulates *miR-27a, miR-20a*, and *miR-17-5p*, resulting in the apoptosis of pancreatic cancer cells [89].

## 4. Bioavailability of dietary components

The anticancer effects of dietary components like EGCG, curcumin, and resveratrol have been reported in a wide variety of cancers [33, 34, 90, 91]. Clinical trial results showed that the prostate cancer risk declined with increasing frequency, duration, and quantity of green tea consumption, and the dose-response relationships were also significant, suggesting that EGCG is effective against prostate cancer [33, 34]. Curcumin and resveratrol, either alone or in combination with other agents, have demonstrated their potential effects against colorectal cancer, pancreatic cancer, breast cancer, prostate cancer, multiple myeloma, lung cancer, oral cancer, and head and neck squamous cell carcinoma [90-91].

Along with laboratory-based results, some clinical trials have demonstrated the chemopreventive and chemotheputic effects of dietary components. However, most of the clinical trials of these components such as EGCG, curcumin, resveratrol, and genisten as anticancer agents are poor bioavailability, raising the debate of the *practical* application of *in vitro* results in physiological states [51, 92, 93]. Clinical studies of these promising molecules demonstrated their low bioavailability, which are hindered by poor water solubility and absorption, as well as rapid metabolism and clearance [51, 92, 93]. The formation of sulphate and glucuronide conjugated by intestine and liver also reduced the bioavailability of some phenolic compounds [51, 93]. Thus, continuing research on these dietary components is needed to provide some possible solutions to overcome these problems. To increase the bioavailability, longer circulation, higher permeability, and resistance to metabolic processes of dietary components, a variety of approaches have been developed, including synthesis of analogues, nanoparticles, liposomes, micelles, and phospholipid complexes [94-96]. The combination use of adjuvants also serves as a useful strategy to improve the bioavailability of dietary components. Recent studies showed that the administration of curcumin or resveratrol with piperine significantly improve their bioavailability through the inhibition of glucuronidation, elevating their concentrations in plasma [97, 98].

## 5. Conclusion and future perspectives

Based on the studies mentioned, it is clear that these dietary components act on different epigenetic targets leading to epigenetic modifications and execute anticancer activities. Current studies also showed that dietary components used in combination with traditional chemotherapy creates potential synergistic effects, including reverting chemotherapy resistance, overcoming the side effects from chemotherapy, and increasing chemotherapy sensitivity, which are all very important for the successful treatment of cancer patients. Insight understanding of the global patterns of epigenetic modifications by dietary components in cancer will necessarily help to develop better strategies to prevent and cure cancer. Therefore, sufficient preclinical data is required for the better understanding of the epigenetic targets and pathways altered by these dietary components to increase the efficacy of their anticancer properties. Additional clinical studies are also needed to analyze the safety profile of dosages, the routes of administration, tissue distribution, as well as bioavailability alone, and in combination with other chemotherapeutic agents in order to obtain the maximum beneficial effects of these dietary components as anticancer agents. Despite these challenges, persistent research on dietary components will offer more epigenetic targets and promising strategies for theprevention and treatment against cancers in the future.

## Abbreviation

AITC: allyl isothiocyanate
BITC: benzyl isothiocyanate
COMT: catechol-*O*-methyltransferase
CpG: cytosinephosphate-guanine
DNMTs: DNA methyltransferases
EGCG: epigallocatechin-3-gallate
EZH2: enhancer of zeste homolog 2
H3K4me1: Monomethylation of histone H3 at lysine 4
H3K4me3: trimethylation of histone H3 at lysine 4
H3K9me3: trimethylation of histone H3 at lysine 9
H3K20me3: trimethylation of histone H3 at lysine 20
H3K27me3: trimethylation of histone H3 at lysine 27
HATs: histone acetyl transferases
HDACs: histone deacetylases
ITCs: isothiocyanate
miRNAs: microRNAs
PITC: phenethyl isothiocyanate
SAH: *S*-adenosyl-_L_- homocysteine
SAM: *S*-adenosyl-methionine
